# Evaluation of Field Map and Nonlinear Registration Methods for Correction of Susceptibility Artifacts in Diffusion MRI

**DOI:** 10.3389/fninf.2017.00017

**Published:** 2017-02-21

**Authors:** Sijia Wang, Daniel J. Peterson, J. C. Gatenby, Wenbin Li, Thomas J. Grabowski, Tara M. Madhyastha

**Affiliations:** ^1^Institute of Diagnostic and Interventional Radiology, Shanghai Jiao Tong University Affiliated Sixth People’s HospitalShanghai, China; ^2^Department of Radiology, University of WashingtonSeattle, WA, USA; ^3^Department of Neurology, University of WashingtonSeattle, WA, USA

**Keywords:** EPI distortion correction, B0 field mapping, symmetric normalization registration, diffusion tensor imaging (DTI), reliability

## Abstract

Correction of echo planar imaging (EPI)-induced distortions (called “unwarping”) improves anatomical fidelity for diffusion magnetic resonance imaging (MRI) and functional imaging investigations. Commonly used unwarping methods require the acquisition of supplementary images during the scanning session. Alternatively, distortions can be corrected by nonlinear registration to a non-EPI acquired structural image. In this study, we compared reliability using two methods of unwarping: (1) nonlinear registration to a structural image using symmetric normalization (SyN) implemented in Advanced Normalization Tools (ANTs); and (2) unwarping using an acquired field map. We performed this comparison in two different test-retest data sets acquired at differing sites (*N* = 39 and *N* = 32). In both data sets, nonlinear registration provided higher test-retest reliability of the output fractional anisotropy (FA) maps than field map-based unwarping, even when accounting for the effect of interpolation on the smoothness of the images. In general, field map-based unwarping was preferable if and only if the field maps were acquired optimally.

## Introduction

Diffusion imaging is a widely-used technique to examine white matter microstructure *in vivo*. Echo planar imaging (EPI), introduced by Mansfield (1977), has become the dominant method for the acquisition of diffusion-weighted images, which are commonly fit to a tensor model as Diffusion Tensor Imaging (DTI). However, EPI causes geometric distortions (Jones and Cercignani, 2010) that stem from the inhomogeneity of the underlying B0 field, which is in turn due to the varying magnetic susceptibilities of air, bone and tissue. Susceptibility-induced EPI distortions alter anatomical fidelity of the images, making anatomically accurate measurements more difficult, and complicate multimodal investigations that integrate data from EPI and non-EPI images.

B0 Field mapping techniques (Jezzard and Balaban, 1995; Wan et al., 1997) can be used to reduce EPI distortion by “unwarping” the images. Unwarping using field mapping requires that we obtain phase images at two different echo times (Reber et al., 1998). Using these two-phase images, one can calculate the degree of EPI distortion present along the phase-encode direction of the EPI images, and then apply an equal and opposite pixel-shift, thereby unwarping the distortions in the original images.

Another approach to correcting EPI distortions at acquisition is the “blip-up blip-down” method introduced by Chang and Fitzpatrick (1992), which requires the acquisition of the same image twice, with reversed phase-encoding gradients. The distortions in each image are identical, but in opposite directions. By applying a specialized symmetric registration with transformations only allowed along the phase-encode direction, we can warp these images to each other to “meet in the middle”, and thereby obtain distortion-corrected images with no signal loss. This method is implemented in FSL’s “topup” program (Andersson et al., 2003).

These two methods must be implemented at the time of acquisition. However, many valuable data sets have been collected and released to the community that have not been acquired with field maps nor with reversed phase-encoding gradients (Zuo et al., 2014).

For this reason, researchers have been motivated to examine correction of distortions using non-linear registration techniques (Andersson et al., 2003; Wu et al., 2008). Non-linear co-registration with the Advanced Normalization Tools (ANTs; Avants et al., 2008) has been used to correct EPI distortions by calculating a nonlinear warp to a non-EPI acquired structural image. The authors found that nonlinear registration increased measures of functional connectivity in a way that suggested the presence of meaningful signal, but the results were different than correction using a field map.

In this article, we examined the question of how well nonlinear co-registration with ANTs corrects distortions as compared to a field map in two different data sets acquired at different sites. While this investigation was conducted in the context of DTI, the results might generalize to fMRI studies that employ unwarping of EPI distortions.

Accurate unwarping is especially important considering that it is an early processing step that may interact with subsequent analytical steps in unexpected ways that may have a large impact on the results of an investigation (Madhyastha et al., 2014). One way of examining whether the distortion correction is accurate or not is to examine the similarity of the fractional anisotropy (FA) map obtained after tensor fitting (assessed using mutual information (MI)) to the structural T1 image. Another is to quantify within- and between-session scan-rescan reliability of distortion correction methods in the context of a complete analysis pipeline, exploiting the idea that in the ideal acquisition scenario, with no variability due to distortion, we would obtain the same DTI statistics in repeated scans. In this article, we compared the reliability of field map-corrected images to nonlinear registration-corrected images after processing using Tract-Based Spatial Statistics (TBSS; Smith et al., 2006), a popular DTI analysis method. We also visualized the difference between FA values acquired at both time points across the entire brain.

## Materials and Methods

### Participants

Our study data came from two data sets, which we label Udall and Boekel. **Udall**: DTI data were acquired as part of a larger study of Parkinson’s disease (Madhyastha et al., 2015). For this study, 23 Parkinson’s patients and 16 controls (mean age = 64.59, SD = 10.53; 16 females) had acceptable quality DTI data for two acquisitions. **Boekel**: The Boekel data set includes data from 32 undergraduate psychology students (mean age = 22.50, SD = 3.22; 17 females) who were recruited from a previous 43-participant magnetic resonance imaging (MRI) study (Boekel et al., 2015). Two subjects from the original data release were omitted from this study because the face shearing used to de-identify subjects caused significant data loss in the T1 and/or field map magnitude images. These data are available for download at NITRC (David, 2006; Boekel et al., 2017). For the Udall data set, the data was collected under a protocol approved by the local University of Washington institutional review board and all participants gave their written informed consent. For the Boekel data set, the data collection protocol was approved by the local ethics committee at the University of Amsterdam (Boekel et al., 2015). All participants gave their written informed consent prior to scanning. The study was approved by the University of Washington review board.

Table 1 shows the demographic data for these subjects. We conducted an independent-sample *t*-test to compare the ages of Udall and Boekel participants. There was a significant difference in the ages for Udall (*M* = 64.59, SD = 10.53) and Boekel participants (*M* = 22.50, SD = 3.22); *t*_(69)_ = 21.76, *p* < 0.001. Participants in the Udall data set are significantly older than the Boekel participants. We used a Chi-square test to compare the percentage of males in each group. There was no significant difference in the percentage of males in each group (*X^2^_(1,N = 71)_* = 1.03, *p* = 0.31).

**Table 1:** **Demographics of Sample**.

	Udall	Boekel
Demographics
*N*	39	32
Age at Scan	64.59 (10.53)	22.50 (3.22)
Sex (number males)	23 (59%)	15 (47%)

### MRI Data Acquisition

In the Udall study, DTI and T1 weighted images were collected on a Philips Achieva 3T scanner at the University of Washington with a 32-channel head coil. The Udall diffusion acquisition was optimized for high angular resolution. The DTI pulse sequence parameters were: 75 2 mm slices acquired with no slice gap and in an ascending temporal slice order, matrix size = 128 × 128, FOV = 256 × 256 × 150 mm, giving a 2 × 2 × 2 mm isotropic voxel size. TR = 10.8 s, TE = 93.5 ms, flip angle = 90°, and a total of 64 *b*-vectors, distributed evenly across a half-sphere, with *b* = 3000 s/mm^2^, and one *b* = 0 s/mm^2^ image. The parallel acceleration factor (SENSE) was 2. Total acquisition time was 14.2 min. The *B*_0_ field map was collected with matching geometry for use in unwarping EPI distortions due to magnetic field inhomogeneity (Jezzard and Balaban, 1995). The field map acquisition was a 3D interleaved dual echo gradient echo pulse sequence, with the following parameters: matrix size = 256 × 256 × 75, FOV = 256 × 256 × 150, TR = 10 ms, TE1 = 2.25 ms, TE2 = 3.25 ms. This bounded the unaliased frequency offsets at ±500 Hz. A sagittal T1-weighted 3D MPRAGE was collected to allow for registration of the DTI images, using the following parameters: 176 slices, matrix size = 256 × 256 × 176, FOV = 256 × 256 × 176 mm, TI = 1100 ms, TE = 3.49 ms, *T* = 7.46 ms, Turbo-Field echo (TFE) factor = 225, flip angle = 7°, shot interval = 2530 ms, and a SENSE factor = 2.

The parameters for the Boekel DTI acquisition are described in Boekel et al. (2015). Briefly, DTI and T1-weighted images were collected on a 3T Philips Achieva XT at the University of Amsterdam with a 32-channel head coil. For each subject, four repetitions of a single shot DTI scan were obtained using the following parameters: 60 2 mm slices acquired with no slice gap and in an ascending temporal slice order, matrix size = 112 × 112, Field of View (FOV) = 224 × 224 × 120, giving a 2 × 2 × 2 mm isotropic voxel size. TR = 7.5 s, TE = 86 ms, flip angle = 90°, and a total of 32 non-colinear b-vectors, distributed evenly across a half-sphere, with *b* = 3000 s/mm^2^, and one *b* = 0 s/mm^2^ image. The parallel acceleration factor (SENSE) of 2. The field map acquisition was a 3D interleaved dual echo gradient echo pulse sequence, with the following parameters: matrix size = 128 × 104 × 128, FOV = 256 × 208 × 256 mm, TR = 11 ms, TE1 = 3 ms, ΔTE = 5 ms. This bounded the unaliased frequency offsets at ±200 Hz. An axial 3D T1-weighted anatomical scan was also acquired with a 3D gradient echo scan with using the following parameters: matrix size = 240 × 188 × 220, FOV = 240 × 188 × 220 mm, TE = 3.8 ms, TR = 7.46 ms, TFE factor = 154, flip angle = 8°, shot interval = 2375 ms, and SENSE factors = 2.5 LR, 2 FH.

We used only the first of the four repetitions of the DTI scan for each session in this analysis. Because the second T1 measurements were dropped for a subset of the Boekel subjects to optimize the total scan duration, we used the first T1 scan for both time points for the Boekel data set.

In the Udall data set, the time between the two DTI acquisitions was 2–3 weeks. In contrast, in the Boekel scanning was completed within a single day; the time between the two scan sessions was 2–4 h.

### MRI Processing

Diffusion data were processed using in-house bash scripts written by DP that invoked FMRIB (Oxford Center for Functional MRI of the Brain) Software Library (FSL) version 5.0.9 programs and produced quality assurance images. These scripts are available at https://github.com/danjonpeterson/dti_preproc. The specific version of the scripts used for this article is version 1.0. Detailed descriptions of the parameters used by all processing steps are given in Supplemental Materials.

**Figure 1:**
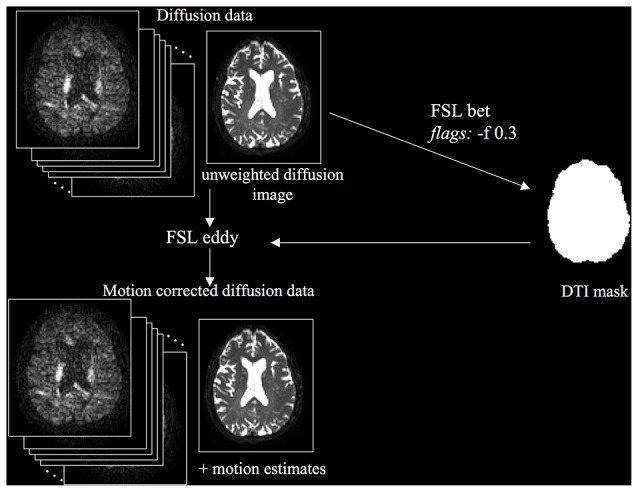
**Diffusion Tensor Imaging (DTI) preprocessing workflow**.

The first step was to correct the diffusion data for motion and eddy-current induced distortions (Figure 1). The unweighted diffusion image was extracted and skull stripped using FSL BET (Brain Extraction Tool) to create a DTI mask. Then, diffusion data were corrected for motion and eddy-current induced distortions using FSL’s “eddy”. Motion estimates, produced using FSL’s rmsdiff command, were used to compute the mean absolute root mean squared (RMS) displacement and mean relative RMS displacement. We analyzed these motion parameters using paired and unpaired *t*-tests, because motion can have a significant impact on the quality and reliability of diffusion MRI data (Yendiki et al., 2014).

**Figure 2:**
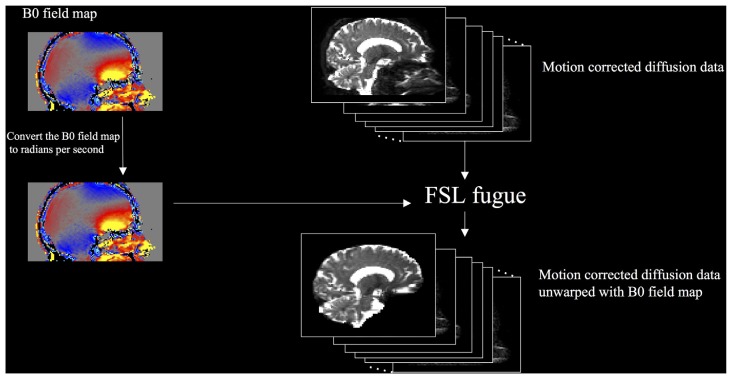
**DTI field map unwarping workflow**.

We compared two methods of performing DTI unwarping. The first used a B0 field map (Jezzard and Balaban, 1995) to compensate for the distortions (field map unwarping), and the second used nonlinear registration (large deformation diffeomorphic metric mapping) implemented as Symmetric Normalization (SyN) by ANTs (Avants et al., 2011) version 2.1.0 release candidate 3 (nonlinear registration unwarping). Field map unwarping (Figure 2) was performed using FMRIB’s Utility for Geometrically Unwarping EPIs (FUGUE). First, the B0 field map was converted to radians per second by multiplying by 2^*^π. FUGUE uses the field map values to determine the pixel shift of the motion-corrected diffusion data in the phase-encode direction, correcting for distortion.

**Figure 3:**
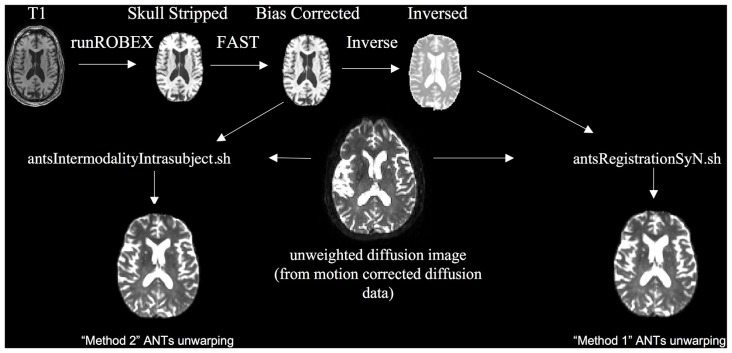
**Nonlinear registration unwarping, implemented using Advanced Normalization Tools (ANTs)**.

To evaluate the scenario where no field map was acquired (Figure 3) we used ANTs SyN nonlinear registration of the unweighted diffusion image to a bias corrected (using FSL FAST) and skull-stripped (using ROBEX, a robust, machine learning-based brain extraction system (Iglesias et al., 2011)) T1 image. We found that ROBEX worked well across both data sets to create an accurate skull stripped image for registration. We performed this registration using two methods. The first method, modeled after EPI motion correction (Huntenburg et al., 2014) was to invert the contrast on the bias corrected and skull stripped T1 image and use the antsRegistrationSynN.sh script to register the B0 image to the inverted T1 image [Method 1]. The second approach was to use the antsIntermodalityIntrasubject.sh script to register the B0 image to the original bias corrected and skull stripped T1 image [Method 2]. Both ANTs approaches use the MI criterion to align the unweighted diffusion image to the T1 image (Huntenburg, 2014; Huntenburg et al., 2014). Method 1 tunes the underlying registration parameters for images that are from different subjects but with similar contrast characteristics. Method 2 tunes the underlying registration parameters for images collected on the same subject, but across image modalities with different contrast characteristics. See “Supplemental Materials” for more details on this pipeline. After visually checking the registrations, we applied the transformation to all volumes of the DTI images.

After unwarping using either the field map or nonlinear registration implemented by SyN in ANTs, we fit the DTI tensor using FSL’s DTIFIT (Basser et al., 1994). We used weighted least-squares fitting to fit the tensor and output DTI scalar images. FA maps obtained using nonlinear registration unwarping were down-sampled to 2 mm.

**Figure 4:**
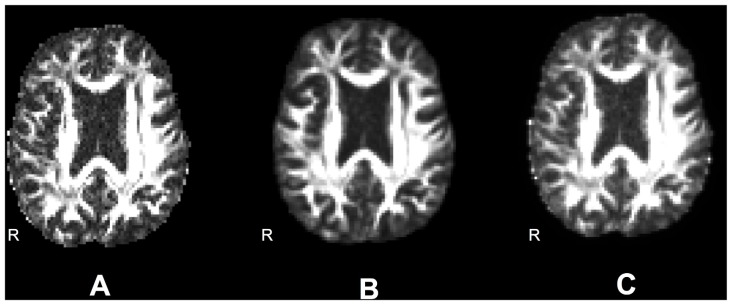
**Differences in smoothing. (A)** Fractional anisotropy (FA) images unwarped according to an acquired field map (smoothness is 4.64). **(B)** FA images unwarped according to nonlinear registration to a structural image (smoothness is 6.76). **(C)** FA images unwarped according to an acquired field map and smoothed to match the smoothness of the nonlinearly warped image (smoothness is 6.73).

FA maps obtained using nonlinear registration unwarping were smoother than those obtained using field map unwarping because of resampling in the registration steps. To separate the effects of smoothing from the effects of unwarping technique, we created a smoothed field map unwarped FA data set as follows. We estimated the smoothness of the nonlinear registration unwarped images using the Analysis of Functional Neuroimaging (AFNI; Cox, 1996) 3dFWHMx utility, which estimates smoothness of the image, or the autocorrelation of neighboring voxels. This is measured as the Full Width at Half Maximum (FWHM) of a Gaussian approximation to the spatial autocorrelation function. We next measured the smoothness of the field map unwarped images to that FWHM estimate using AFNI 3dBlurToFWHM, which iteratively re-smooths a data set until it has the given FWHM in the specified dimensions. After unwarping using ANTs, the estimated mean FWHM smoothness across all dimensions was 10.27 mm for the Boekel data set, and 10.48 mm for the Udall data set. Figure 4 shows an example axial slice of a single subject’s FA map using field map unwarping (A), after nonlinear registration unwarping (B), and after matching the smoothing of the field map unwarping to the nonlinear registration unwarping (C).

This processing pipeline was written in GNU Make, using the approach described in Askren et al. (2016). Makefiles describing the workflow are available from the corresponding author.

### Evaluation of Unwarping

We evaluated unwarping using two different techniques. One way to quantify unwarping is to compare the unwarped image to an undistorted image. To operationalize this approach, we quantified the MI between the computed FA map and the skull-stripped T1 image. MI, or the statistical dependence between the intensities of corresponding voxels in two images, is an important measure of image similarity that is often used to evaluate registration methods. MI should be maximized when images are aligned (Maes et al., 1997). MI is calculated using marginal distributions derived from a joint histograms of the two images (Avants et al., 2011) using the MeasureImageSimilarity program from ANTs. MI is by definition a positive value, but the metric output by ANTs is sign-inverted so that it can be minimized; therefore, the method that has the lowest value of this metric (the highest MI) can be interpreted as having the least distortion in the FA map relative to the T1. We used this metric to choose the ANTs registration that best aligned the FA map to the T1 (of the two methods that we tried shown in Figure 3) for each individual. We report the negative of the ANTs reported metric, the MI metric, where higher MI is better.

Another way to evaluate the effect of unwarping methods is to consider how different unwarping techniques affect subsequent analytic power. We operationalize this by evaluating the reliability of DTI scalar statistics in a realistic analysis setting using TBSS. TBSS projects all subjects’ FA data onto a mean FA tract skeleton that represents the centers of all tracts common to the group. TBSS has gained popularity for its ease of use and improved alignment of white matter as compared to other voxel-wise analysis methods (Jones and Cercignani, 2010). We assessed reliability by running TBSS (using default parameters (Jenkinson, 2013)) on the FA maps at each session computed using no unwarping, field map unwarping and nonlinear registration unwarping. We calculated voxel-wise statistics on the final TBSS skeleton using Pearson’s correlation coefficient (*r*) and the Intra class Correlation (ICC) coefficient to assess the reliability of FA statistics across sessions as in prior work (Madhyastha et al., 2014). To compute the ICC, we used a two-way mixed effects model with absolute agreement (McGraw and Wong, 1996). We determined whether there was an overall significant difference in reliability between voxels in a skeleton by using a Mann-Whitney *U*-test, a nonparametric test of the null hypothesis that the distributions of the reliability coefficients in the skeleton are identical using data unwarped using different techniques.

We also examined a measure of reliability across the entire brain, including areas where we expect signal dropout, by calculating the absolute value of the difference in FA maps for each subject at each time point after registering to the structural image obtained at the first-time point using rigid-body registration. We then used ANTs SyN registration to transform the structural image to standard space, and applied this nonlinear transform to the FA difference maps. In this way we computed a “mean FA difference map” for each study and each method. We used random permutation testing to conduct a paired *t*-test to determine where the difference in longitudinal measurements using field map unwarping was smaller or larger than the measurements using ANTs unwarping. We implemented this *t*-test using FSL randomize, with threshold-free cluster enhancement (Smith and Nichols, 2009) to identify clusters without specifying an arbitrary threshold and correction for multiple comparisons by controlling the family-wise error (FWE) rate. This allowed us to partially separate effects of registration from the effects of unwarping to visualize spatial differences in the reproducibility of measures using the two unwarping methods.

## Results

We compared the mean absolute RMS displacement and mean relative RMS displacement across time points and studies using paired sample *t*-tests. For Udall, the mean absolute RMS displacement was 0.51 mm and the mean relative RMS displacement was 0.89 mm. There was no significant difference between the mean absolute RMS motion (*t*_(38)_ = 0.20, *p* = 0.84) and mean relative RMS motion (*t*_(38)_ = 0.82, *p* = 0.42) between the two-time points. For Boekel, the mean absolute RMS displacement was 0.20 mm and the mean relative RMS displacement was 0.55 mm. There was no significant effect for the mean absolute RMS displacement (*t*_(31)_ = 0.78, *p* = 0.44) and mean relative RMS displacement (*t*_(31)_ = 0.45, *p* = 0.66) between the two-time points. But the mean absolute RMS displacement and mean relative RMS displacement are both higher in Udall than in Boekel (*t*_(140)_ = 11.61, *p < 0*.001) and mean relative RMS motion (*t*_(140)_ = 4.93, *p* < 0.001).

Table 2 shows the MI of the FA maps resulting from field map unwarping and nonlinear registration unwarping to the bias corrected and skull stripped T1. Estimates across different sessions are quite stable (they are the same to within two significant digits, and not shown). For Udall, field map unwarping is the best. No unwarping is better than nonlinear registration unwarping (*t*_(77)_ = 17.14, *p* < 0.001) and field map unwarping is better than no unwarping (*t*_(77)_ = −4.15, *p* < 0.001) and nonlinear registration unwarping (*t*_(77)_ = −15.89, *p* < 0.001). For Boekel, however, we saw that nonlinear registration unwarping is the best. Nonlinear registration is better than no unwarping (*t*_(63)_ = −25.38, *p* < 0.001) and field map unwarping (*t*_(63)_ = 26.16, *p* < 0.001). No unwarping is better than field map unwarping (*t*_(63)_ = 2.88, *p* =* 0*.005).

**Table 2:** **Mean mutual information (higher is better) of FA and T1 images across all subjects using different unwarping techniques**.

	Udall time 1^1^		Boekel time 1	
	*M*	*SD*	*M*	*SD*
No unwarping	0.47	0.03	0.39	0.02
ANTs (method 1)	0.45	0.03	0.43	0.03
ANTs (method 2)	0.44	0.03	0.41	0.03
ANTs (best of method 1 and 2)^2^	0.45	0.03	0.43	0.03
Field map unwarping	0.48	0.04	0.39	0.02

*Method 1 uses antsRegistrationSynN.sh and Method 2 uses antsIntermodalityIntrasubject.sh. Note: ^1^Results for time 1 are identical to within two significant digits to results from time 2, indicating that methods worked similarly across both samples. ^2^The highest MI is computed on a per-subject level, so it is theoretically possible for this value to be larger than the mean for either method*.

Table 3 shows the Pearson correlation and ICC values calculated without unwarping, using field map unwarping, nonlinear registration unwarping, and field map unwarping after smoothing to match the field map unwarping. In general, nonlinear registration unwarping produced the highest reliability of voxels in the TBSS skeleton. Some form of unwarping improved reliability over no unwarping, despite the fact that TBSS uses nonlinear registration implemented by fnirt and skeleton projection to align white matter. For both Boekel and Udall, there was a significant improvement in reliability from simply smoothing the field map unwarped image. The magnitude of this improvement was, in general, larger than the magnitude of the improvements from unwarping. Consistent with differences in quality of unwarping using the field map for the two acquisitions as measured using MI, nonlinear registration unwarping for Boekel resulted in a similar percentage of voxels with a significant correlation or ICC value, and a higher mean, than field map unwarping with smoothing. In contrast, the Udall results show a somewhat lower percentage of voxels with a significant correlation or ICC value for nonlinear registration unwarping than field map unwarping with smoothing, and a resulting smaller improvement in reliability with nonlinear registration unwarping. Mann-Whitney *U* tests showed that the mean ICC values were significantly different for all methods and data sets at *p* < 0.001.

**Table 3:** **Reliability of different methods for unwarping (in the TBSS pipeline)**.

		Pearson correlation			ICC		
		Mean in the skeleton	Percentage of voxels (*p* < 0.05) in the skeleton	Mean among voxels (*p* < 0.05) in the skeleton	Mean in the skeleton	Percentage of voxels (*p* < 0.05) in the skeleton	Mean among voxels (*p* < 0.05) in the skeleton
Udall	No unwarping	0.66	0.81	0.69	0.66	0.82	0.67
	Field map unwarping unsmoothed	0.70	0.79	0.72	0.70	0.80	0.71
	Field map unwarping smoothed	0.77	0.82	0.76	0.76	0.82	0.75
	Nonlinear registration unwarping	0.80	0.79	0.78	0.80	0.78	0.78
Boekel	No unwarping	0.62	0.80	0.66	0.61	0.83	0.65
	Field map unwarping unsmoothed	0.64	0.82	0.68	0.64	0.85	0.67
	Field map unwarping smoothed	0.68	0.86	0.72	0.67	0.88	0.70
	Nonlinear registration unwarping	0.77	0.87	0.79	0.77	0.87	0.77

**Figure 5:**
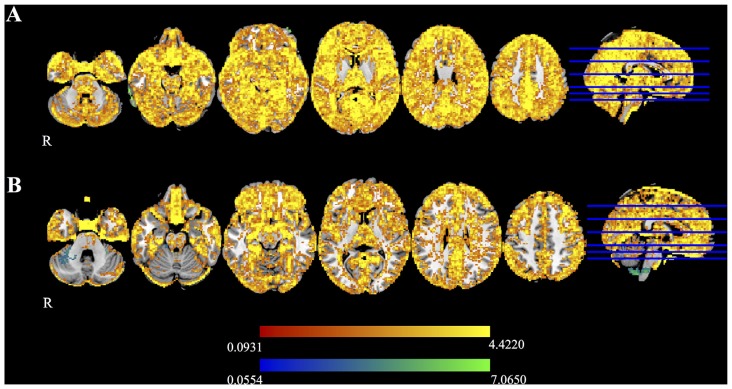
**Difference maps for the (A)** Udall and **(B)** Boekel data sets, as measured by the difference in FA at two time points. Red-yellow voxels show where nonlinear registration to a structural image produces smaller differences between time points, and blue-light blue voxels indicate that the field map-based unwarping produces smaller differences between time points. All highlighted voxels are family-wise error (FWE)-corrected for multiple comparisons (*p* < 0.05).

Figure 5 shows the mean difference in FA calculated between time 1 and time 2 for Udall and Boekel. Red-yellow shades show areas where FA maps obtained using registration unwarping are more similar between time points, and blue shades show areas where FA maps obtained using field map unwarping are more similar between time points. All results are FWE-corrected at *p* < 0.05. In most areas of the brain, the registration unwarping shows greater similarity between time points than field map unwarping.

## Discussion

In this study, we found that distortion correction of diffusion data using the SyN algorithm implemented in ANTs resulted in higher reliability, both as measured after processing using TBSS and by examining mean difference scores (Figure 5), than using a field map for unwarping. This suggests that unwarping using nonlinear registration may be a reasonable choice, especially when no explicit method of distortion correction is available.

In contrast, results obtained simply by comparing MI scores were equivocal. Using MI scores themselves we would conclude that unwarping using nonlinear registration using ANTs could potentially be worse than doing no unwarping. However, in real analyses MI is not normally an outcome metric, and because the downstream effects of processing steps can be profound, it is important to consider each step in the context of the analysis one plans to conduct.

Specifically, we note that “secondary” processing effects on the data, such as smoothing due to resampling, can have a larger impact on downstream measures than the quality of the correction itself. For example, smoothing increases the reliability of TBSS results, in many cases more than the effect of distortion correction. Although the MI of the structural image and the FA map is larger with field map unwarping than with nonlinear registration unwarping, nonlinear registration unwarping still has the highest reliability in the TBSS pipeline and according to the mean difference in FA statistics across time points. This occurs because the mean FA maps at each time point end up being more similar to each other than to the structural image. Although smoothing increases the reliability of the data and decreases noise, it may also decrease sensitivity to detect important differences. This tradeoff must be considered when developing an analytic pipeline.

The difference in results obtained using the same unwarping pipeline on two different field map and diffusion data acquisitions reminds us that the quality of field map unwarping is only as good as the quality of the field map. The static magnetic field within a scanner can be easily measured using standard pulse sequences. One commonly used sequence acquires two gradient echoes with slightly differing TEs: for any collection of spins within a voxel, its phase difference between the two echoes is linearly proportional to its field offset. However, a certain amount of care is needed when setting up a field-mapping sequence. Firstly, the acquisition volume should be set to be the same FOV in all three directions as the DTI scans. Secondly, the shimming method should be the same for the field-map. These two settings are important as usually a scanner performs a field shimming step as part of its pre-scan procedure, which optimizes the shim over the requested FOV (or a manually defined region). If this region for the field-map is different from the region for the DTI scans, or the shimming method is different, then the actual shim will be different, and hence the field-map will not be measuring the field that exists for the DTI scans. Thus, of course, any subsequent use of this different field-map to correct the distortion in the DTI scans will be invalid.

The differences between the Boekel and Udall field maps are that the Boekel field map has a large ΔTE, and that there is a field of view difference between the diffusion scan and the field map in the Boekel dataset. The parameter difference is a suboptimal design, and this is reflected in the lower reliability after unwarping.

To summarize, our recommendations for best practices for addressing susceptibility artifact correction using field maps are as follows:

At Acquisition: To obtain a useful field map (i.e., one that measures the same magnetic field that is experienced by the diffusion weighted image, with the highest fidelity):

1. The Field of View, number of slices, slice thickness (and slice gap if using a 2D sequence), and angulation must be the same as the diffusion MRI sequence. We prefer a 3D scan for greater signal to noise ratio (SNR), taking care to ensure that the 3D slice thickness is equal to the DTI slice thickness plus any slice gap. This ensures that the shimming process for the field map is over the same volume as the diffusion weighted image.
2. The first echo should be set to the shortest possible time, with the delta TE to 1 ms—which allows for a ±500 Hz range before phase-wrapping occurs, while still providing sufficient “dynamic range” for the observed frequency offsets.
3. The TR can be set to the shortest available, or lengthened, if a magnitude image with some T1 contrast is desired for use in an image registration pipeline is desired (see point 6).
4. The shimming method should be the same as the diffusion weighted scan(s), and preferably based on a pencil-beam and/or volume shimming method.
5. The field map should ideally be acquired after the diffusion-weighted scan(s), and any pre-scan steps set to a mode in which the shim values are taken from the previous diffusion scan, and not recalculated (points 1 and 4 help assure that no re-shimming is performed for the field map).
6. The acquired matrix should be set to the same resolution as the diffusion-weighted scan, or an integer multiple thereof. We have found that using a 256 × 256 matrix for the field map, compared with 128 × 128 matrix for the diffusion weighted scan, allows for the magnitude images from the field map to be used as a helpful intermediate step in registration from the DTI scans to high-resolution structural scans.

For pre-existing data sets:

1. If a field map exists and conforms to the specifications above, it can be used to correct distortions. This approach should give the best correspondence of the unwarped diffusion image to anatomical images independent of subsequent processing steps.
2. If there is no field map, or if it is suboptimal, use nonlinear registration with ANTs to correct distortion.

Note that other acquisition-based methods for distortion correction, such as blip-up blip-down acquisition methods (Chang and Fitzpatrick, 1992), may be superior to field map unwarping, but this comparison is beyond the scope of this article.

There are some limitations to our study. We used reliability data sets collected for purposes other than for this study. If we were designing a study with the sole purpose of exploring the efficacy of different unwarping techniques, we would subject the same individuals to different acquisition techniques designed to correct EPI distortions. We would also collect non-EPI acquired T2 contrast images to optimize registration. We would ensure that field maps were optimal with respect to the diffusion MRI scan. There are also other pipelines that we could have evaluated for unwarping and other parameters for nonlinear registration unwarping, which may be more effective for different data sets. Our evaluation focused on MI and reliability of FA maps. Different analyses may result in different conclusions.

However, our findings with these two data sets show that there is a clear benefit to distortion correction, and that correction with nonlinear registration using ANTs is a reasonable technique, which can be used with some confidence in the absence of field maps or field-reversed DTI acquisition.

## Author Contributions

SW, TJG, TMM and WL: study conception and design; SW, DJP, JCG, WL, TJG and TMM: analysis and interpretation of data and drafting of manuscript; DJP, TJG and TMM: critical revision of the manuscript.

## Conflict of Interest Statement

The authors declare that the research was conducted in the absence of any commercial or financial relationships that could be construed as a potential conflict of interest.
