# Supplementary material for: Evaluation of Field Map and Nonlinear Registration Methods for Correction of Susceptibility Artifacts in Diffusion MRI
Source: Front Neuroinform. 2017 Feb 21;11:17. doi: 10.3389/fninf.2017.00017 (PMC5318394; doi:10.3389/fninf.2017.00017)
Supplement: Supplementary file 1 [file DataSheet_1.pdf]

MRI Processing Details

DTI PREPROCESSING AND FIELDMAP UNWARPING

DTI preprocessing and fieldmap unwarping were executed using a set of preprocessing scripts that are publically available on github. These scripts also generate HTML quality-control reports. The specific version of these scripts used in this research can be found here:
https://github.com/danjonpeterson/dti_preproc/releases/tag/v1.0
This version will not change, even if the main branch is updated. Further documentation on options to these scripts are are available through the README of this repository

DTI preprocessing (Figure 1)
Dti preprocessing, including the calls to bet and eddy, were executed through `motion_correct.sh` in the `dti_preproc` scripts.

FSL bet

bet performs skull stripping. The fractional intensity (flag: -f) was 0.3, which is reduced from the default 0.5. This results in a more liberal brain mask, which is appropriate for b0 images, as *bet* was developed for structural images.

Example command:

```
bet dti_b0.nii.gz dti_b0_brain.nii.gz -f 0.3
```

FSL eddy

eddy corrects eddy-current induced distortions. The input mask was created using *bet*, as above

The Acquisition parameter file was as follows:

```
0 1 0 0.072
```

This is a “dummy” uninformative acquisition parameter file, as we are just using *eddy* for motion-correction, and not for unwarping (which would required blip-up, blip-down data).

Beyond this, default parameters were used, some of which are as follows:
- number of iterations (--iter): 5
- final interpolation (--resamp): spline with jacobian modulation

Further documentation can be found here:
https://fsl.fmrib.ox.ac.uk/fsl/fslwiki/eddy/UsersGuide#List_of_parameters

Example commandl (through dti_preproc scripts):

```
motion_correct.sh -k dti.nii.gz -b dti.bval -r dti.bvec -o dti -M nodif_mask.nii.gz
```

Unwarping with b0 field map (Figure 2)
Fieldmap unwarping is performed using FSL *Fugue.* This is called by the `unwarp_fieldmap.sh` script.

FSL fugue

A skull-stripped magnitude image was created by running *bet* with no flags on the fieldmap magnitude image

Default parameters were used, some of which are as follows:
- unwarping direction (--unwarpdir) was in the y direction

Fugue was re-run again with --nokspace to reverse-warp the magnitude image for comparison with the distorted b0, for evaluating the quality of the unwarp.

Further documentation can be found here:
https://fsl.fmrib.ox.ac.uk/fsl/fslwiki/FUGUE/Guide#FUGUE

Example commandl (through dti_preproc scripts):
```
unwarp_fieldmap.sh -k dti/mc_dti.nii.gz -f DTI_B0_phase.nii.gz -m DTI_B0_mag.nii.gz -M
DTI_B0_mag_brain_mask.nii.gz -o dti -p DTI_B0_mag_brain_mask.nii.gz -t 0.567 -e 93.46
```

ANTS unwarping (Figure 3)
ANTs unwarping was implemented using Makefiles available from the first author. The Makefiles call several programs to perform skull stripping, bias estimation, image inversion, and ANTs registration. ANTs registration is performed using two scripts are supplied by the authors of ANTs, which are interfaces (or 'wrappers') to the more general *antsRegistration* utility. In effect, they simplify the setting of options to antsRegistration for specific applications. More information can be found here: https://github.com/stnava/ANTsDoc/raw/master/ants2.pdf

runROBEX
ROBEX (RObust Brain EXtraction) is a robust skull-stripping program for structural T1 images, which is based on a machine learning approach with a random forest classifier. More documentation is available here:
https://sites.google.com/site/jeiglesias/ROBEX/documentation

(ROBEX takes no parameters by design)

Example command:
```
runROBEX.sh T1.nii.gz T1_robex.nii.gz
```

FAST
FAST (FMRIB's Automated Segmentation Tool) is an image segmentation tool for T1 images. However, in this investigation we use only the bias-correcting function in FAST, rather than using it to segment tissue types. RF inhomogeneities can introduce low spatial-frequency intensity variations in the T1 image, which can confound nonlinear registration. Bias correction in FAST is based on an expectation-maximization approach. More documentation can be found here:
https://fsl.fmrib.ox.ac.uk/fsl/fslwiki/FAST

Parameters supplied were as follows:
- bias corrected image (-B) was added to the output

Example command:
```
fast -B T1_robex.nii.gz
```

Image Inversion
For ANTs unwarping “Method 1”, the contrast in the bias-corrected T1 image was inversed following the approach of Huntenberg et al (2014). This is done so that the contrast characteristics of the image become similar to that of the B0, where CSF is high-intensity and brain parenchyma is low-intensity. This was accomplished with image arithmetic using *fslmaths* and *fslstats,* preserving the image range in the anatomical image.

In mathematical form:

$$T1_{inv} = \left[\frac{T1_{max} - T1_{min}}{b0_{max} - b0_{min}}\right] \cdot \left(-T1_{orig} + T1_{max}\right)$$

Where:
-$T1_{inv}$ is the outputted inverted image
-$T1_{orig}$ is the input bias-corrected T1 image
-$T1_{max}$ and $T1_{min}$ are the maximum and minimum values in the (skull-stripped) T1 image
-$b0_{max}$ and $b0_{min}$ are the maximum and minimum values in the (skull-stripped) b0 image

antsIntermodalityIntrasubject.sh
This script, part of ANTs, sets up an *antsRegistration* call that is appropriate for finding a warp between images collected on the same subject, but across imaging modalities with different contrast characteristics. This was used for “Method 2”

Parameters supplied were as follows:
- Dimensionality (-d) was 3, for MRI volume data
- Transform type (-t) was 2, for an initial rigid registration and a subsequent small deformation nonlinear registration
- The bias-corrected T1 was the ‘fixed’ image, and the skull-stripped b0 was the ‘moving’ image

Example commandl:
```
antsIntermodalityIntrasubject.sh -d 3 -i dti_b0_brain.nii.gz -r T1_robex_restore.nii.gz -x
T1_robex_restore.nii.gz -w template -o B0toT1SmallWarp -t 2
```

antsRegistrationSyN.sh
This script, part of ANTs, sets up an *antsRegistration* call that is appropriate for finding a nonlinear symmetric warp between images with that are not necessarily collected on the same subject, but across imaging modalities with similar contrast characteristics. This was used for “Method 1”

Parameters supplied were as follows:
- Dimensionality (-d) was 3, for MRI volume data
- Transform type (-t) was ‘s’ for an initial rigid registration, followed by an affine registration, followed by a final nonlinear symmetric normalization
- The inverted T1 was the ‘fixed’ image, and the skull-stripped b0 was the ‘moving’ image

Example command:

```
antsRegistrationSyN.sh -d 3 -f T1_inverse.nii.gz -m dti_b0_brain.nii.gz -o B0toT1 -t s
```
